# Neonatal Diabetes Mellitus Due to Homozygous ZNF808 Mutation Associated With Skeletal Anomalies: A Novel Presentation of Pancreatic Agenesis-3

**DOI:** 10.7759/cureus.103126

**Published:** 2026-02-06

**Authors:** Ali S Alquraishi, Musa M Saad, Syed Rayees

**Affiliations:** 1 Pediatrics, Endocrinology Unit, Armed Forces Hospital Southern Region, Khamis Mushait, SAU; 2 Paediatrics, Armed Forces Hospital Southern Region, Khamis Mushait, SAU

**Keywords:** neonatal diabetes mellitus, pediatrics & neonatology, skeletal deformities, whole-exome sequencing, znf808 mutation

## Abstract

Neonatal diabetes mellitus (NDM) is a rare genetic condition characterized by persistent hyperglycemia presenting within the first six months of life. It is most commonly caused by mutations affecting pancreatic development or β-cell function. ZNF808 is a recently identified gene that plays an essential role in human pancreatic development, with biallelic pathogenic variants causing autosomal recessive pancreatic agenesis.

We report the case of a seven-month-old Saudi male infant with a history of intrauterine growth restriction who presented at 2.5 months of age with severe hyperglycemia and polyuria. Clinical examination revealed congenital skeletal anomalies, including a fixed flexion deformity of the right knee and oligodactyly of the right foot. Whole-genome sequencing identified a homozygous likely pathogenic variant in the ZNF808 gene, confirming a diagnosis of pancreatic agenesis-3.

This case highlights a rare genetic cause of neonatal diabetes and suggests a novel association between ZNF808 mutations and skeletal anomalies, a feature not previously reported. These findings emphasize the importance of early genetic testing in neonatal diabetes, particularly in the presence of congenital anomalies and parental consanguinity.

## Introduction

Neonatal diabetes mellitus (NDM) is a rare disorder presenting with insulin-dependent hyperglycemia within the first six months of life. It is distinct from autoimmune type 1 diabetes and results from mutations affecting pancreatic development, insulin secretion, or β-cell function. NDM may be transient or permanent, with an estimated incidence of 1 in 90,000 to 160,000 live births [1].

More than 30 genes have been implicated in the pathogenesis of NDM, including KCNJ11, ABCC8, and INS. In addition, mutations in transcription factors such as PTF1A, GATA6, PDX1, and RFX6 have been associated with syndromic forms of NDM, frequently accompanied by pancreatic agenesis or hypoplasia [2].

ZNF808 is a primate-specific Krüppel-associated box (KRAB) zinc-finger protein that has recently been shown to be essential for human pancreatic development. Biallelic pathogenic variants in ZNF808 cause autosomal recessive pancreatic agenesis, as first described by De Franco et al. [3].

Here, we report a case of neonatal diabetes mellitus due to a homozygous ZNF808 mutation, presenting with skeletal anomalies and confirmed by whole-genome sequencing.

## Case presentation

A seven-month-old Saudi male infant, born to consanguineous parents, was delivered via elective cesarean section due to fetal distress and intrauterine growth restriction. He was admitted to the neonatal intensive care unit for seven days due to respiratory distress, during which he received empirical intravenous antibiotics and supplemental oxygen. No hypoglycemia, hyperglycemia, or sepsis was documented during this period.

On neonatal examination, the infant was not dysmorphic and had normal cardiovascular findings. Notably, he had a congenital fixed flexion deformity of the right knee and oligodactyly of the right foot, with only four toes visible. He was discharged in good condition.

At 2.5 months of age, the mother observed that her baby was experiencing polyuria for approximately 10 days. She was uncertain about the exact number of nappies changed per day. During this period, the baby was fed 60 to 70 mL by bottle every 2 to 3 hours. Concerned about this symptom, the family sought medical attention at a local hospital. Investigations revealed a markedly elevated blood glucose level, prompting initiation of subcutaneous insulin therapy. The patient was referred to our tertiary center for further workup and evaluation.

Upon presentation, the infant was alert, well hydrated, and hemodynamically stable. His vital signs were as follows: pulse rate of 112 beats per minute, respiratory rate of 32 breaths per minute, and blood pressure of 92/60 mmHg. His weight was 3.7 kg. Capillary blood glucose was 36.3 mmol/L. Venous blood gas analysis showed a pH of 7.40 and bicarbonate (HCO₃⁻) of 23.7 mmol/L, excluding diabetic ketoacidosis (DKA). HbA1C, serum insulin, and C-peptide were assessed (Table 1). Thus, a diagnosis of neonatal diabetes was established.

**Table 1:** Blood workup

Description	Result	Reference range
HbA1c	13.64%	<5.7%
Insulin	2.7 μU/mL	<15 μU/mL
c-peptide	0.38 ng/mL	0.5-2 ng/mL

He was admitted to the pediatric intensive care unit (PICU), and subcutaneous insulin therapy was initiated using insulin Aspart (NovoRapid) and insulin Degludec (Tresiba). To allow for accurate low-dose administration, insulin was diluted with normal saline at a 1:100 ratio. Doses were titrated according to capillary glucose monitoring. After six days of stabilization, the patient was transferred to the pediatric ward in good condition.

Both parents were educated and trained in diabetes care, including insulin injection techniques, glucose monitoring, and hypoglycemia recognition.

The combination of diabetes mellitus presenting in early infancy and associated skeletal abnormalities prompted genetic evaluation. Whole-genome sequencing identified a homozygous likely pathogenic variant in the ZNF808 gene, confirming a diagnosis of autosomal recessive pancreatic agenesis (pancreatic agenesis-3) (Table 2).

**Table 2:** Whole-exome sequencing study SNP: single-nucleotide polymorphism.

Gene	Variant coordinates	Amino acid change	SNP identifier	Zygosity	In silico parameters	Type and classifications
ZNF808	NM_001039886.3:C.1448dup	p.(Tyr483Ter)	N/A	Homozygous	PolyPhen:N/A Align-GVGD:N/A MutationTaster:N/A Conservation_nt Conservation_aa	Nonsense likely pathogenic (class 2)

## Discussion

Neonatal diabetes mellitus is clinically recognized in infants younger than six months who exhibit ongoing hyperglycemia requiring insulin therapy, with the diagnosis subsequently supported by molecular genetic analysis to identify an underlying monogenic etiology. This step is recommended to guide therapeutic decisions.

Neonatal diabetes mellitus due to ZNF808 mutations is exceedingly rare. ZNF808 encodes a KRAB zinc-finger protein involved in transcriptional regulation during embryogenesis and plays a critical role in pancreatic lineage specification and endocrine cell differentiation [3].

Wolcott-Rallison syndrome remains the most common syndromic cause of neonatal diabetes mellitus and results from pathogenic variants in EIF2AK3, leading to impaired endoplasmic reticulum stress responses [4]. In the present case, the combination of early-onset diabetes, skeletal anomalies, and parental consanguinity strongly suggested a monogenic etiology.

In this case, the presence of polyuria, persistent hyperglycemia, and absence of ketoacidosis at 2.5 months of age were consistent with neonatal diabetes mellitus. The additional findings of skeletal anomalies and a history of consanguinity suggested a syndromic and potentially genetic etiology, warranting early genetic evaluation.

Pancreatic agenesis is more commonly associated with mutations in GATA6, PTF1A, and PDX1 [2]. However, ZNF808 has recently emerged as a novel contributor. Unlike some previously reported cases, our patient did not exhibit clinical exocrine pancreatic insufficiency.

Management of neonatal diabetes focuses on metabolic stabilization followed by insulin therapy once enteral feeding is established. Insulin may be administered subcutaneously using multiple daily injections or continuous subcutaneous insulin infusion [5]. Approximately 40% and up to 88% of neonatal diabetes cases caused by KCNJ11 and ABCC8 mutations are responsive to oral sulfonylurea therapy [6-10]; however, pancreatic agenesis necessitates lifelong insulin treatment.

This case adds to the growing body of evidence supporting the role of ZNF808 in pancreatic development and expands the phenotypic spectrum of neonatal diabetes mellitus. Genetic testing, especially whole genome or exome sequencing, remains crucial for early diagnosis and targeted management of monogenic diabetes.

To our knowledge, skeletal anomalies have not been previously described in association with ZNF808-related neonatal diabetes. Our patient exhibited a fixed flexion deformity of the right knee and oligodactyly of the right foot. While other genetic forms of pancreatic agenesis, such as those caused by GATA6, PDX1, or PTF1A, have occasionally been associated with extrapancreatic features, the ZNF808 gene has not been linked to limb anomalies. This raises the possibility of an expanded phenotypic spectrum or a potential modifier gene effect. Further case accumulation and functional studies are warranted to clarify this observation.

## Conclusions

This case underscores a rare presentation of neonatal diabetes mellitus caused by a homozygous mutation in ZNF808. The presence of skeletal anomalies and consanguinity further supported a genetic basis. This case contributes to the understanding of the phenotypic spectrum of ZNF808-associated pancreatic agenesis and suggests that skeletal anomalies may be part of its clinical presentation. Recognition of such atypical features can aid in early diagnosis and broaden current genotype-phenotype correlations.

Early genetic diagnosis not only confirmed the underlying cause but also informed clinical management and genetic counseling. Recognition of such rare mutations expands our understanding of the molecular basis of pancreatic agenesis and emphasizes the importance of including ZNF808 in genetic panels for neonatal diabetes.
